# Sustainable Valorization of *Aloe vera* Skin: Development of a New Emulgel with Anti-Inflammatory Properties

**DOI:** 10.3390/molecules31152735

**Published:** 2026-08-06

**Authors:** Jessica Ceramella, Elisabetta Bruno, Maria Stefania Sinicropi, Francesca Romana Lupi, Vincenzo Sicari, Antonio Gattuso, Carlos Fernandes, Daniel Chavarria, Filomena Conforti, Giancarlo Statti, Mary Fucile, Carlo Maria Scornajenghi, Fernanda Borges, Domenico Iacopetta, Domenico Gabriele

**Affiliations:** 1 Department of Pharmacy, Health and Nutritional Sciences, University of Calabria, Via Pietro Bucci, 87036 Rende, Italy; jessica.ceramella@unical.it (J.C.); filomena.conforti@unical.it (F.C.); giancarlo.statti@unical.it (G.S.); mary.fucile@unical.it (M.F.); domenico.iacopetta@unical.it (D.I.); 2 Department of Information, Modeling, Electronics and Systems (D.I.M.E.S.), University of Calabria, Via Pietro Bucci, Cubo 39C, 87036 Rende, Italy; elisabetta.bruno@unical.it (E.B.); francesca.lupi@unical.it (F.R.L.); domenico.gabriele@unical.it (D.G.); 3 Department of Agriculture, University “Mediterranea” of Reggio Calabria, Via dell’Università, 89125 Reggio Calabria, Italy; vincenzo.sicari@unirc.it (V.S.); antonio.gattuso@unirc.it (A.G.); 4 MedInUP/RISE-Health, Pharmacology and Therapeutics Unit, Department of Biomedicine, Faculty of Medicine, University of Porto, Alameda Prof. Hernâni Monteiro, 4200-319 Porto, Portugal; carlos.fernandes@med.up.pt; 5 Department of Maternal-Infant and Urological Sciences, Sapienza University of Rome, Via del Policlinico 155, 00161 Rome, Italy; carlomaria.scornajenghi@uniroma1.it; 6 RISE-Health, Department of Chemistry and Biochemistry, Faculty of Sciences, University of Porto, Rua do Campo Alegre, s/n, 4169-007 Porto, Portugal; fborges@fc.up.pt

**Keywords:** *Aloe vera*, Gastro-Esophageal Reflux Disease, xanthan emulgel, agricultural waste

## Abstract

*Aloe vera* is a renowned phytotherapeutic plant characterized by a highly dynamic chemical profile and endowed with a broad spectrum of biological activities. Industrial processing of *A. vera* focuses almost exclusively on the inner parenchyma (AG), discarding the epidermal tissue (AS) as waste. This study addresses the valorization of this byproduct through a comparative evaluation of the phytochemical and biological profiles of hydroalcoholic extracts obtained from both AG and AS. In vitro studies revealed that AS extract exhibited better antioxidant and anti-inflammatory activities than AG and good wound-healing properties, driven by its rich content of anthraquinones and polyphenols. Both extracts were successfully incorporated into a mucoadhesive xanthan gum matrix to engineer an advanced emulgel with suitable viscosity for prolonged mucosal adherence in Gastro-Esophageal Reflux Disease (GERD) management. Rheological profiling against commercial references confirmed that the xanthan-AS formulation provided comparable rheological properties, fully preserving the extract’s anti-inflammatory activity. Ultimately, this study highlights the pharmaceutical potential of *A. vera* skin, offering a sustainable strategy to transform an industrial byproduct into an innovative formulation for potential GERD management.

## 1. Introduction

*Aloe vera* (L.) Burm. f. (*Aloe barbadensis* Miller), belonging to the *Asphodelaceae* family, is one of the most ancient and celebrated medicinal plants in history. Documented by ancient civilizations as the "plant of immortality," it has been utilized for millennia as a cornerstone of phytotherapy due to its extraordinary therapeutic properties [1]. The chemical profile of *A. vera* (*Av*) is highly dynamic, influenced by cultivation conditions, harvest timing, climate, and extraction methodologies [2]. Traditionally, the pharmaceutical and cosmetic industries have focused on the inner mucilaginous gel, which is composed of approximately 99.5% water. The remaining solid fraction contains over 200 bioactive compounds, including proteins, lipids, amino acids, vitamins, enzymes, and a diverse range of water- and lipid-soluble polyphenols [3]. This complex matrix is responsible for numerous biological activities: the structural polysaccharides, such as glucomannans, modulate immune responses and accelerate tissue repair, while phenolic compounds, including flavonoids and anthraquinones, provide significant antioxidant, anti-inflammatory, and antimicrobial properties [4]. Specifically, anthraquinones like aloin and aloe-emodin play a crucial role in modulating the inflammatory response by blocking the arachidonic acid pathway, reducing cyclooxygenase-2 (COX-2) and inducible nitric oxide synthase (iNOS) levels, or interfering with the expression of peroxisome proliferator-activated receptor-gamma (PPARγ) and 11β-hydroxysteroid dehydrogenase (11β-HSD1) [5]. In LPS-activated macrophages, aloe metabolites significantly reduced the production of pro-inflammatory cytokines (TNF-α and IL-12) and nitric oxide (NO) by inhibiting iNOS expression and suppressing the MAPK signaling pathway [6]. In addition, wound healing and pro-angiogenic properties of this plant extract are attributed to acetylated acemannans that stimulate the expression of the Vascular Endothelial Growth Factor (VEGF) and Type I collagen [4]. During industrial processing, the inner gel is separated from the outer green skin. This rind constitutes over 30% of the total leaf weight and is typically discarded as agro-industrial waste [7]. However, the recent literature suggests that the aloe skin is also a potent source of bioactive molecules, containing significantly higher concentrations of anthraquinones, polysaccharides, polyphenols, and essential minerals (iron, zinc, calcium, and manganese) than the gel itself [7].

These multi-target biological activities, combining potent antioxidant and anti-inflammatory effects with tissue-repair capabilities, make *Av* extracts particularly promising for managing widespread conditions, including GERD, characterized by the retrograde flow of gastric contents into the esophagus, persistent heartburn, and a lower quality of life for millions of individuals worldwide [8]. Indeed, oral administration of aloe syrup effectively reduces GERD symptoms, such as heartburn, regurgitation, and nausea, with minimal side effects [9].

Current GERD pharmacological interventions typically rely on bismuth-containing agents, histamine H2-receptor antagonists, and various antacid formulations. Among these, alginate-based systems (such as Gaviscon^®^) are widely recognized for their mechanical action; they react with the stomach acid environment to create a durable, floating “raft” that acts as a physical barrier against acid reflux [10,11]. Literature data even suggest that integrating *A. vera* into alginate-based rafts yields cohesive, viscoelastic cross-linked networks with enhanced therapeutic potential [12]. However, beyond this mechanism, other therapeutic approaches focused on mucosal protection and topical healing of the injured esophagus. To this end, bioadhesive polymeric matrices offer an ideal vehicle for targeted, gastroprotective drug delivery [10]. While the majority of literature reports alginate-based formulations, fewer studies have also explored the applications of pectin, xanthan gum, or raw psyllium fibers. In particular, xanthan gum is a bacterially derived pentasaccharide stabilizer that shows significant promise in GERD therapy, due to its mucosal-protective properties. The clinical efficacy of xanthan gum with *Av* gel is well-established by commercial medical devices such as GerdOff^®^. In this formulation, xanthan gum serves as a structuring and mucoadhesive matrix that retains *Av*, hyaluronic acid, and chondroitin sulfate on the esophageal mucosa, providing a sustained protective barrier and promoting tissue repair in GERD patients.

Building upon these observations, the present study focuses on the development of a novel xanthan gum-based gel formulation designed for GERD management, optimized to achieve the necessary viscosity for prolonged gastroesophageal mucosal adherence. Crucially, while conventional anti-reflux strategies and commercial reference standards like GerdOff^®^ exclusively rely on the inner gel of the plant, discarding hundreds of tons of leaf skin as agricultural waste, the core novelty of this work lies in the valorization and biological exploitation of the *Av* epidermal skin, turning an agro-industrial byproduct into a high-value resource.

Herein, a hydroalcoholic mixture (EtOH:H_2_O 8:2) was employed to extract and comprehensively compare the bioactive profiles of both the inner parenchyma/gel (AG) and the epidermal tissue/skin (AS), specifically antioxidant capacity, anti-inflammatory activity, and wound-healing properties. Driven by the promising bioactive superiority of the skin extract, the AS fraction was successfully incorporated into a xanthan gum matrix. The resulting formulation was rheologically characterized, compared with commercial standards (Gaviscon^®^ and GerdOff^®^), and evaluated for its capacity to preserve and deliver the extract’s intrinsic anti-inflammatory property. Ultimately, this study proposes a high-value sustainable strategy to transform agricultural waste into an advanced pharmaceutical formulation for potential GERD management.

## 2. Results and Discussion

### 2.1. Extraction and Yields

The *Av* leaves were processed to obtain two distinct extracts, designated AG (inner parenchyma/gel) and AS (epidermal tissue/skin). Both were extracted using a hydroalcoholic solution (EtOH/H_2_O 8:2 *v*/*v*) *via* maceration, applying two distinct protocols: for the gel, dynamic maceration was performed, and the solvent volume was calibrated to minimize the phytocomplex’s exposure to oxidative and thermal stress, as it is known that heat treatments significantly reduce overall antioxidant capacity [13]. In contrast, the skin was subjected to a classical maceration process to ensure complete recovery of its metabolites. This approach is consistent with data indicating that the leaf peel is the primary site of accumulation for phenolic compounds and lipophilic antioxidants [14], with concentrations of specific compounds, such as α-tocopherol, reaching levels up to ten times higher than those found in the gel [15]. The extraction process yielded 2.32% for the gel and 2.60% for the peel (*w*/*w* _fresh raw material_). Although high-energy or high-temperature methods (e.g., Soxhlet extraction, heat-assisted extraction, or prolonged microwave-assisted extraction) typically yield higher yields, their use can significantly reduce antioxidant activity. This loss, which can reach up to 57%, is mainly due to the thermal degradation of polyphenols and sensitive vitamins [15,16], confirming the choice of a room-temperature maceration process to preserve the biological integrity of the phytocomplex.

### 2.2. Characterization

*Av* is a plant of notable pharmaceutical, nutraceutical, and cosmetic interest due to the presence of several metabolites including enzymes, phenolic compounds, vitamins, organic acids, and polysaccharides. Several studies have investigated its biological activities, including anti-inflammatory, anti-arthritis, and wound-healing properties [17,18,19]. The main phenolic compounds identified in *Av* extracts from both AG and AS extracts are reported in Table 1. Results showed statistically significant differences in phenolic content between the two extracts, with higher concentrations generally present in AS, except for aloe emodin, which was found in traces in AS.

The most abundant class of compounds detected was anthraquinones, mainly aloins: aloin A and B, also known as barbaloin and isobarbaloin. In AS extract, aloin A was found at a concentration of 4.10 mg g^−1^ compared to 0.042 mg g^−1^ in AG; similarly, aloin B was quantified at 2.992 mg g^−1^ in AS and 0.023 mg g^−1^ in AG. The main transformation and degradation products of barbaloin are considered aloin B and aloe emodin [20].

Another characteristic phenolic identified compound was aloeresin D, an aloesin derivative, also found in higher amounts in AS. 3-O-*p*-Coumaroylquinic acid is a compound belonging to the family of quinic acids and their derivatives, characterized by a cyclic structure containing four hydroxyl groups and a carboxylic group. This compound was found in many *Aloe* species [21,22], but it is still under investigation. 3-O-*p*-Coumaroylquinic acid was found to be strongly correlated with antioxidant activity, particularly in the ABTS assay, due to the presence of numerous hydroxyl groups, which act as effective hydrogen donors, and the conjugate structure, which facilitates electronic delocalization and stabilization of phenoxyl radicals. It also showed a significant correlation with the inhibitory activity against α-glucosidase, presumably due to the presence of key functional groups, such as OH groups and conjugated double bonds, known to modulate interaction with digestive enzymes.

Therefore, 3-O-*p*-Coumaroylquinic acid emerges as an important bioactive polyphenolic compound with a double role: on the one hand, it contributes to the antioxidant activity of phytocomplexes; on the other hand, it participates in the modulation of carbohydrate metabolism through enzymatic inhibition, making it a potential candidate for nutraceutical and functional applications [23].

The phenolic compounds identified are considered key markers of Aloe [24], further supporting its functional and pharmaceutical value.

The higher phenolic content in AS was further confirmed by the determination of the total phenolic content (TPC) and the total flavonoid content (TFC), as shown in Table 2. Consistent with the profile of the individual markers, AS showed a higher polyphenol and flavonoid content than AG, confirming, overall, the compositional differences observed for the individual phenolic and anthraquinone markers. The higher TPC and TFC values observed in the AS compared to the AG are consistent with previous studies on *Av*, which consistently describe the peel fraction as richer in phenolic compounds and flavonoids than the gel, with peel extracts reporting TPC values around 16.88 mg GAE/g and TFC values around 32.08 mg QE/g [25], while peel extracts reach TPC values ranging from 86.5 to 125.8 mg GAE/g under optimized extraction conditions [7]. This compositional distribution reflects the anatomical localization of phenolic and anthraquinone compounds, which are concentrated primarily in the outer peel and in the latex-containing pericyclic cells located beneath the epidermis, while the gel consists mainly of water and polysaccharides, with a relatively dilute phenolic fraction.

### 2.3. Biological Activity

#### 2.3.1. ROS Scavenging Assays

Redox balance is involved in the regulation of different cellular activities and signaling pathways, but an uncontrolled and prolonged Reactive Oxygen species (ROS) production may contribute to the development of chronic inflammatory responses and damage subcellular components (e.g., DNA, proteins, and lipids), resulting in the onset and/or progression of several pathological conditions [26]. Thus, restoring intracellular redox balance represents a promising strategy for promoting, overall, cellular homeostasis. To investigate the potential antioxidant activity of the extracts AG and AS, different ROS scavenging assays using the following radicals, 2,2-diphenyl-1-picrylhydrazyl (DPPH^•^), 2,2′-azino-bis(3-ethylbenzothiazoline-6-sulfonic acid) radical cation (ABTS^•+^), and galvinoxyl (GO^•^), were performed. Moreover, the iron (Fe^2+^) chelation capacity of the extracts was evaluated. Table 3 resumes the obtained results, where it is possible to see that the AG extract was unable to scavenge all the used radicals. Additionally, the AS extract displayed lower radical scavenging activity (IC_50_ = 619.67 µg/mL) against DPPH^•^ radical compared to the reference antioxidant Trolox (IC_50_ = 6.46 µg/mL). Similar results were obtained for ABTS^•+^ (AS IC_50_ = 56.90 µg/mL *versus* Trolox IC_50_ = 4.97 µg/mL) and GO^•^ (AS IC_50_ = 530 µg/mL *versus* Trolox IC_50_ = 1.26 µg/mL). However, AS showed a fair activity in the Fe^2+^ chelation assay performed using ethylenediaminetetraacetic acid (EDTA) as a positive control, with IC_50_ values of 66.89 and 0.77 µg/mL, respectively. This is an important feature, because it is well-known that some biological processes are catalyzed by enzymes that need metals but, in other cases, the exacerbated presence of certain heavy metals may impair the normal activity of different biomolecules [27].

#### 2.3.2. Anti-Inflammatory Properties

The anti-inflammatory activity of AG and AS extracts was investigated through the Griess assay, adopting the murine macrophages RAW 264.7. Indeed, macrophages can produce nitric oxide (NO), together with other pro-inflammatory cytokines (e.g., TNF-α and interleukins), when treated with the bacterial lipopolysaccharide (LPS). It is known that NO is synthetized by the nitric oxide synthase (NOS) enzymes and acts as a second messenger in inflammatory processes [28]. Under an inflammatory stimulus, the inducible NOS (*i*NOS) produces NO from L-arginine. However, its overexpression and/or dysregulation are involved in chronic inflammation, cancer, and neurodegeneration [29].

RAW 264.7 cells were incubated with different concentrations of AG and AS extracts (500, 600, 750, 800, and 1000 µg/mL) or the positive control indomethacin (Ind) (70, 140, and 280 µg/mL), a nonsteroidal anti-inflammatory drug (NSAID). LPS was employed at a concentration of 1 µg/mL for inducing NO production in RAW 264.7 cells. Figure 1 shows the obtained results after 24 h of treatment. Ind reduced NO production in a dose-dependent manner; compared to LPS-only treated cells (CTRL), it decreased NO production in 10, 16, and 36% at 70, 140, and 280 µg/mL, respectively (light green columns). A dose-dependent decrease in NO production was also recorded with AS treatment, with decreases of 15–54% between 600 and 1000 µg/mL. Conversely, no effect was observed under AG treatment. This outcome may result from different factors [30]; however, in this study, a lower concentration of some bioactive compounds with anti-inflammatory properties could be the most plausible explanation. Indeed, Table 1 supports this hypothesis, together with the dramatic difference in antioxidant activity between AG and AS extracts previously determined (see Section 2.3.1 and Table 3).

Then, cell viability was also evaluated using the MTT assay, performed under the same experimental conditions of the Griess assay, to exclude any cytotoxic effect of the tested extracts in RAW 264.7 cells. Results show the absence of a significant cell viability reduction compared to the CTRL (dark green), except for Ind at 280 µg/mL, which produced a slight but significant (about 10%) cell viability reduction. From these results, it is evident that AS exhibits good anti-inflammatory properties, although with lower potency when compared to Ind. Interestingly, AS at 1000 µg/mL reduced the NO production by half in stimulated macrophages, without affecting cell viability. Ind, at the concentrations of 140 and 280 µg/mL (seven and 3.5 times lower than the highest AS dose), reduced NO production by 16 and 36%, but also produced a 10% cell viability reduction at the highest dose, meaning that the observed NO reduction is doubtlessly affected by cell death. Overall, the disclosed properties of AS extract and the lack of cytotoxicity on the murine macrophages used in these assays suggest a high potential as an anti-inflammatory agent.

##### Effect of AS Extract on iNOS Expression and NF-κB Nuclear Translocation

Since the reduced NO production by stimulated RAW 264.7 macrophages observed after AS extract exposure can depend on a direct inhibition of *i*NOS activity but also on a reduced amount of the expressed protein [31], *i*NOS expression was investigated by the means of immunostaining assays, under the same experimental conditions used for the Griess assay. Briefly, RAW 264.7 cells were exposed to DMSO (vehicle, negative control), LPS or co-treated with LPS (1 µg/mL) and Ind (positive control) or AS, for 24 h. Then, cells were further processed as described in the Section 3. Figure 2a resumes the obtained outcomes where it is possible to see an increased expression of *i*NOS in LPS-stimulated macrophages compared to the DMSO-treated cells (Figure 2a, CTRL, panels B, red fluorescence). Conversely, the co-treatment with Ind produced a decrease in *i*NOS protein expression by approximately 56%, as expected and already reported [32,33]. AS extract treatment led, as well, to a 46% reduction in *i*NOS expression (Figure 2a, AS, panels B). Fluorescence quantification was also reported (Figure 2b). Overall, these results indicate that the inhibitory activity of the AS extract against *i*NOS is accompanied by its ability to reduce LPS-induced *i*NOS expression in RAW 264.7 cells.

It is noteworthy that *i*NOS expression can be regulated by different factors, particularly *via* the transcriptional activity of NF-κB, which is a pivotal regulator in cell biology [34]. The inactive NF-κB form has a cell cytoplasm localization and, upon stimulation and activation, it translocates into the nucleus, where it regulates the expression of target genes that encode for proteins involved in different processes, including inflammation, proliferation, apoptosis and so on [35]. The intracellular localization of NF-κB in RAW 264.7 cells was investigated by immunofluorescence assays under the same experimental conditions adopted for the Griess assay and the semiquantitative *i*NOS detection. As shown in Figure 3, DMSO-treated cells exhibited a predominantly cytoplasmic localization of NF-κB (negative control, panel B), whereas, after LPS treatment, it migrates inside the cell nuclei, as indicated by the white arrows (Figure 3, LPS, panel B), as further confirmed by the overlay of red (NF-κB) and blue (nuclei) fluorescence signals in Panel C. LPS + Ind co-treatment partially reversed NF-κB activation, as deducible by its decreased nuclear localization (Figure 3, panels B, white arrows, and C, merge), as well as the co-treatment LPS + AS, meaning that AS was also able to decrease the NF-κB nuclear translocation (Figure 3, panels B, white arrows, and C, merge). These results indicate that AS exposure reverses the LPS-induced activation of NF-κB and supports the reduction in *i*NOS expression previously observed.

#### 2.3.3. Evaluation of Scratch Closure

*Av* gel is well known for its several beneficial effects in skin disorders. For instance, it can reduce skin fragility, improve skin integrity and moisture retention, prevent ulcers, and contribute to skin healing [1]. To investigate the potential healing activity of the two *Av* extracts, AG and AS, we employed a well-known, low-cost, and validated in vitro model of wound healing, namely the scratch assay, using an epithelial mammary gland cell line, i.e., MCF 10A cells. After being processed as described in the Section 3, MCF-10A cells were treated with AG or AS at a concentration of 25 µg/mL or vehicle (DMSO, CTRL) and observed at time 0 (immediately after scratching) and after 24 and 48 h of treatment. The adopted concentration was experimentally deduced from preliminary experiments that allowed the lowest concentration to produce the best effect. MTT assays revealed that IC_50_ values for AG and AS were, in both cases, higher than 1000 µg/mL. Figure 4A,B resume the obtained outcomes, in which it is possible to see that, after 24 h, AG produced a better cell migration rate and a wound closure of about 85% compared to time 0, whereas AS behavior was similar to that of vehicle-treated cells (CTRL), amounting to about 64% of wound closure. However, after 48 h, both extracts induced an almost total wound closure (about 99%) while in vehicle-treated cells, the closure was still partial (about 77%). These observations indicate that both extracts produced a beneficial effect on epithelial cell migration and proliferation and suggest a high potential for the treatment of wounds, burns, and ulcers. More importantly, to the best of our knowledge, this effect was already known for AG but not for AS, highlighting an exploitable function of an *Av* byproduct.

### 2.4. Formulation Development and Rheological Characterization

#### 2.4.1. Formulation Development

Based on the promising antioxidant, anti-inflammatory, and wound-healing properties described above, a novel xanthan gum emulgel incorporating the AG and AS extracts was developed. This formulation was designed for the management of GERD and optimized to achieve the necessary viscosity for prolonged gastroesophageal mucosal adherence. To develop an optimal mucoadhesive delivery system, blank emulgels were prepared with varying concentrations of xanthan gum (0.4%, 0.45%, and 0.5% *w*/*w*) and designated as XG0.4, XG0.45, and XG0.5, respectively. This gradient was investigated to evaluate the precise impact of the polymeric matrix concentration on the structural and rheological behavior of the emulgels, with the ultimate goal of identifying the formulation that best matches the performance of some used commercial benchmarks (Gaviscon^®^ and GerdOff^®^). Both products are widely prescribed for the symptomatic management of GERD and are characterized by a semi-solid, gel-like consistency, making them ideal rheological benchmarks. Gaviscon^®^ is an alginate-based raft-forming antiacid containing sodium alginate, sodium bicarbonate, and calcium carbonate, which precipitates into a mechanical barrier upon contact with gastric acid. In contrast, GerdOff^®^ is designed as a topical mucosal protectant; its formulation combines hyaluronic acid, chondroitin sulfate, and *A. vera* inner gel within a bioadhesive xanthan gum matrix to shield the esophageal mucosa and facilitate tissue repair.

Based on preliminary screening, the XG0.4 matrix was selected as the most promising formulation for the incorporation of the extracts due to its balanced rheological profile. Consequently, AG and AS extracts were loaded into this specific vehicle at a concentration of 10 mg/mL, yielding the formulations XG0.4_10AG and XG0.4_10AS. The extract concentration of 10 mg/mL represents the maximum incorporation threshold for these systems; indeed, preliminary trials showed that higher concentrations led to heterogeneous and granular emulgels, compromising physical stability.

#### 2.4.2. Rheological Characterization of Benchmark Samples

The rheological characterization of the selected reference materials was performed to reproduce their rheological properties.

The results of the stress sweep tests in terms of storage (G′) and loss (G″) moduli *versus* shear stress are reported in Figure 5. As shown by the profiles, the developed carriers and the commercial benchmark products, Gaviscon^®^ and GerdOff^®^, display a broad and well-defined linear viscoelastic range (LVR). Within this linear region, the limiting value of G′ extracted to describe the modulus of the raft when the applied stress approaches zero (G′_0_, equal to the value of G′ at the lowest adopted stress) for our vehicle aligns closely with those of the reference products, indicating a comparable structural firmness and network density at rest. Furthermore, the limit of the LVR representing the critical stress at which structural breakdown begins occurs at highly similar stress thresholds across all tested samples (ranging from 1 to 10 Pa), confirming equivalent structural behavior under deformations.

The results of the frequency sweep test in terms of $G^{\prime }$, $G^{\prime \prime }$ (a) and phase angle *δ* (b) versus frequency of oscillation (ω) are reported in Figure 6, and the complex modulus G* and *δ* at 1 Hz are reported in Table 4.

For both samples, storage modulus $G^{\prime }$ is higher than loss modulus $G^{\prime \prime }$ over almost the entire frequency range, indicating a predominantly elastic behavior; this behavior agrees with what was found by [36]. However, at high frequencies, the moduli tend to overlap in the case of Gaviscon^®^, whereas, for GerdOff^®^, a crossover between $G^{\prime \prime }$ and $G^{\prime }$ appears, suggesting a transition toward a more viscous-dominated response. In addition, Gaviscon^®^ exhibits higher moduli values compared to GerdOff^®^ in the investigated range of frequency, indicating a more consistent system also confirmed by the higher value of the complex modulus $G^{*}$ reported in Table 4. Regarding the phase angle, Gaviscon^®^ shows lower values in the low-frequency region, reflecting a more pronounced structuration compared to GerdOff^®^; however, at 1 Hz, the values are almost the same. The increase in $G^{\prime \prime }$ and *δ* with the frequency suggests that the viscous contribution becomes more relevant at higher frequencies.

As far as flow behavior is concerned, the obtained parameters of the flow curve model (Equation (4)) are reported in Table 4. Gaviscon^®^ exhibited a higher consistency index compared with GerdOff^®^, indicating a greater resistance to deformation in the investigated shear rate range and thus confirming SAOS results, at the same flow index.

#### 2.4.3. Rheological Characterization of Xanthan Gum Formulation

The results of the rheological characterization of the XG emulgels are shown in Figure 6 in terms of $G^{\prime }$ and $G^{\prime \prime }$ (a), and *δ* (b) as a function of frequency, and in comparison with reference samples. Furthermore, $G^{*}$ and δ at 1 Hz are reported in Table 4.

The results show, as expected, an increase in the moduli with increasing XG concentration, a behavior already reported in the literature [37]. For all samples, the storage modulus $G^{\prime }$ was higher than the loss modulus $G^{\prime \prime }$ over the entire frequency range investigated, and both moduli exhibited a weak dependence on frequency. The phase angle (δ) remained below 45° and was nearly constant across the frequency range, indicating a predominantly solid-like behavior.

The flow curves of the samples are reported in Figure 7. All formulations exhibited shear-thinning behavior. The viscosity data were fitted using the power-law model (Equation (4)), and the obtained parameters are reported in Table 4. An increase in the consistency index (k) was observed with increasing xanthan gum concentration, whereas the flow index (n) decreased, indicating a more pronounced shear-thinning behavior.

Compared with the reference samples, $G^{\prime }$ exhibited a similar frequency dependence, whereas a different trend was observed for $G^{\prime \prime }$. In particular, $G^{\prime \prime }$ showed a weaker dependence on frequency, suggesting the presence of a more structured network. Based on the results obtained from the oscillatory tests, among the investigated samples, XG0.4 exhibited rheological properties more similar to those of the reference samples. In particular, its complex modulus was closer to that of Gaviscon^®^, and its phase angle showed the closest agreement with the references compared to the other XG samples. Similarly, viscosity measurements showed that XG0.4 displayed flow properties comparable to those of the references. Therefore, this formulation was selected for the incorporation of the *Aloe* extract.

#### 2.4.4. Rheological Characterization of Xanthan Gum Formulations Loaded with Aloe Extracts

Frequency sweep tests and flow curves were performed on the extract-loaded samples to assess whether the incorporation of the *Aloe* extracts significantly affected the rheological properties of the emulgel. The results are reported in Table 4. The presence of the extracts resulted in a slight decrease in the complex modulus and an increase in the phase angle, indicating that both the strength and the network structure are lower than those of the XG0.4 emulsion gel.

In fact, the decrease in the elastic modulus after the addition of *Aloe* extracts can be attributed to a weakening of the xanthan gum network. The soluble components present in the extract may interfere with intermolecular interactions between xanthan chains, thereby reducing the overall network strength and viscoelastic properties, as commonly reported for modified xanthan systems [38]. Indeed, previous studies have shown that alterations in the molecular structure of xanthan can lead to a decrease in its viscoelastic properties [39].

Nevertheless, the formulations enriched with extracts exhibited a complex modulus more similar to that of GerdOff^®^ and a higher degree of structure (inversely proportional to the phase angle) compared to the unloaded sample, which was more similar to that of the reference products.

With regard to flow behavior, the consistency index showed a significant decrease in both samples containing the extracts compared to the sample without the extracts, while the flow index remained statistically comparable.

Moreover, based on the source of the *Aloe* extract (i.e., whether from gel or skin) used to modify the xanthan gum gels, it can be stated that the network of sample XG0.4_10AG is slightly less strong and structured (lower value of G* and higher value of δ) than that produced with the skin (sample XG0.4_10AS), whereas these differences do not appear to be significant in terms of flow properties.

However, in light of all the results presented and discussed, it can be concluded that both of the loaded samples are comparable, in terms of rheological properties, to those of the GerdOff reference sample.

### 2.5. Evaluation of the Anti-Inflammatory Properties of Xanthan Gel Formulation Containing the AS Extract (XG0.4_10AS)

Aiming at evaluating whether the formulation containing the AS extract (XG0.4_10AS) still maintained the already proved anti-inflammatory properties of the AS extract by itself, we employed once again the Griess assay in RAW 264.7 cells. First, we exposed RAW 264.7 cells to three different concentrations of the formulation containing AS (XG0.4_10AS), 500, 800, and 1000 µg/mL or the “empty” formulation (XG0.4) (not containing AS extract), for 24 h. Next, LPS (1 µg/mL) was used as an inducer of NOS-mediated NO formation, and cell viability and NO production were measured, as detailed in the Section 3. Figure 8A,B depict the obtained outcomes. First, compared to cells only stimulated with LPS, both XG0.4 and XG0.4_10AS did not exhibit cytotoxic effects in RAW 264.7 cells, since no reduction in cell viability was recorded at concentrations up to 1000 µg/mL (Figure 8A). Furthermore, a dose-dependent reduction in NO production was recorded for XG0.4_10AS (Figure 8B), indicating that the emulgel preparation process did not alter the anti-inflammatory properties previously demonstrated with the extracts alone (Figure 1). Particularly, XG0.4_10AS produced a reduction in NO production of about 25 and 49% at 800 and 1000 µg/mL, respectively, compared to the LPS-treated cells (CTRL). Conversely, the “empty” formulation XG0.4 did not exert any effect on NO production, under the same experimental conditions. The formulation XG0.4_10AG, containing the AG extract, was also tested but, as expected, did not produce any effect on NO production. In summary, the XG0.4_10AS formulation was demonstrated to exert good anti-inflammatory properties without producing cytotoxic effects in this experimental cell model.

## 3. Materials and Methods

### 3.1. Plant Material and Sample Preparation

Fresh, mature leaves (3–4 years) of *A. vera*, provided by “Azienda Agricola Camasco” (Scalea, Cosenza) in October 2022, were washed with distilled water, and the epidermal tissue (skin) was manually separated from the inner aqueous parenchyma (gel). Both fractions were cut into small pieces and subjected to an extraction process by maceration. Specifically, the gel (233.67 g) was extracted via dynamic maceration in dark condition, using a hydroalcoholic solution (EtOH/H_2_O 8:2 *v*/*v*). The system was kept under continuous agitation for 3 h. The epidermal portion (370 g), on the other hand, was subjected to static maceration at room temperature in a dark container. The material was immersed in a hydroalcoholic solution (EtOH/H_2_O 8:2 *v*/*v*). The procedure involved three consecutive extraction cycles, with the solvent replaced every 48 h. The resulting preparations were combined and dried using a rotary evaporator at 35 °C. The dry extracts obtained were designated AG (from the gel) and AS (from the skin) and stored at 4 °C before being used in subsequent biological tests.

### 3.2. Liquid Chromatographic Conditions

Methanol, ultrapure water, and acetonitrile (UHPLC grade, Carlo Erba, Milan, Italy) were used for chromatographic analysis. 3-O-p-Coumaroylquinic acid, Aloin B, Aloin A, Aloeresin D, Aloe emodin (Sigma Aldrich, St. Louis, MO, USA).

The qualitative and quantitative analysis of polyphenols in aloe samples was performed in an ultra-high-performance liquid chromatography system (UHPLC - PLATINblue system- Knauer, Berlin, Germany). The analysis was conducted injecting 2 μL of properly diluted extract with a flow rate of 0.4 mL/min. The analysis was carried out with a binary pump (A and B), a C18 column (1.8 μm, 100 × 2 mm) set at 30 °C and a Photo Diode Array Detector. The elution gradient used in pump A was acidified water (CH_2_O_2_-pH 3.10), and acetonitrile. The applied gradient was as follows: 95% A for 3 min; 95–60% A up to 15 min; 60–0% A 1 min and finally returning to the original conditions. Quantification was performed with the external standard calibration curve for each compound and results were calculated as mg g^−1^ extract.

### 3.3. Total Phenolic Content (TPC) and Total Flavonoid Content (TFC)

Total phenolic content was determined using the Folin–Ciocalteu colorimetric method: samples dissolved at a concentration of 2 mg/mL (0.2 mL) in a mixture of acetone/methanol/water/acetic acid (40:40:20:0.1) were treated with 1 mL of Folin–Ciocalteu reagent and 1 mL of 7.5% (*w*/*v*) Na_2_CO_3_ solution, followed by incubation at 60 °C for 1 h. Two hours after incubation, the absorbance was measured at 726 nm.

To determine the flavonoid content, 1 mL of sample (2 mg/mL in 80% ethanol) was reacted with 1 mL of 2% AlCl_3_, and the absorbance was measured at 430 nm after a 15 min reaction. Each analysis was performed in triplicate. Quantification was obtained by interpolation using calibration curves constructed with chlorogenic acid (CAE) as the reference standard for polyphenols and quercetin (QE) for flavonoids; the results are expressed as mg of standard equivalents per gram of dry extract [40].

### 3.4. Biological Evaluation

#### 3.4.1. Antioxidant Activity

##### 2,2-Diphenyl-1-Picrylhydrazyl Radical (DPPH^•^) Radical Scavenging Assay

The radical scavenging properties of the *Av* extracts against the 1,1-diphenyl-2-picrylhydrazyl radical (DPPH^•^) were studied as reported [41]. An ethanolic DPPH^•^ solution (6.85 μM) was prepared and subsequently adjusted by dilution to achieve an absorbance value of 0.72 at 515 nm. Six different concentrations of AG or AS extracts (ranging between 10 μg/mL and 1 mg/mL) were tested, adding DPPH˙ solution (180 μL) to the compound solution (20 μL) in triplicate, and the radical scavenging activity was recorded by measuring the absorbance at 515 nm after 30 min. The inhibition percentages (%I_DPPH_) were determined *versus* the initial concentration of DPPH (CTRL, 20 µL of ethanol and 180 µL of ${\mathrm{DPPH}}^{•}$ solution) according to the following Equation (1):

I_DPPH_ (%) = [(ABS_CTRL_ − ABS_sample_)/ABS_CTRL_] × 100 (1)

where ABS_CTRL_ and ABS_sample_ are the absorbance values at 515 nm in the absence and presence of the sample, respectively. The I_DPPH_ percentages were plotted against the respective concentration, and the IC_50_ values were determined using Graph-Pad Prism 9 software (GraphPad Inc., San Diego, CA, USA). Trolox was used as a reference antioxidant.

##### 2,2′-Azino-bis(3-ethylbenzothiazoline-6-Sulfonic Acid) Radical Cation (ABTS^•+^) Scavenging Assay

The 2,2′-azinobis-(3-ethylbenzothiazoline-6-sulfonic acid) radical cation (${\mathrm{ABTS}}^{•+}$) was generated by the addition of aqueous solution of potassium persulfate (2.45 mM final concentration) to the aqueous solution of ABTS (7 mM) and by storage in the dark at room temperature for 16 h [41]. This solution was diluted with methanol to reach the absorbance of 0.72 at 734 nm. A total of 20 μL of the extract’s solutions (six concentrations ranging between 10 μg/mL and 1 mg/mL) were added to 180 μL of ABTS^•+^ radical solution, and the absorbance at 734 nm was read after incubation of 15 min. The inhibition percentages (%I_ABTS_) were determined *versus* the initial concentration of ABTS (CTRL, 20 µL of ethanol and 180 µL of ${\mathrm{ABTS}}^{•+}$ solution) according to the following Equation (2):

I_ABTS_ (%) = [(ABS_CTRL_ − ABS_sample_)/ABS_CTRL_] × 100 (2)

where ABS_CTRL_ and ABS_sample_ are the absorbance values at 734 nm in the absence and present of the sample, respectively. The I_ABTS_ percentages were plotted against the respective concentration, and IC_50_ values (μg/mL) were calculated. Trolox was used as a reference antioxidant.

##### Galvinoxyl (GO^•^) Radical Scavenging Assay

The ${\mathrm{GO}}^{•}$ radical scavenging assay was performed as already described [42]. Six different concentrations (ranging between 10 μg/mL and 1 mg/mL) of extracts were prepared in ethanol and ${\mathrm{GO}}^{•}$ ethanolic solution (5 mM) was prepared and diluted until the absorbance of 1 (428 nm). AG or AS extracts solution (20 µL) were mixed with ${\mathrm{GO}}^{•}$ solution (180 µL) and measured at 428 nm for 30 min. The % radical inhibition was determined by comparing the control (blank, 20 µL of ethanol and 180 µL of ${\mathrm{GO}}^{•}$ solution) to the AG or AS extract solutions, and dose–response curves allowed the IC_50_ values calculation (Graph-Pad Prism 9 software, GraphPad Inc., San Diego, CA, USA). Trolox was used as a reference antioxidant.

##### Iron(II) Chelating Capacity

The extracts were evaluated for the iron(II) chelating properties using the ferrozine method previously reported [42]. Briefly, 20 μM ammonium iron(II) sulfate hexahydrate in a 0.2 M ammonium acetate buffer pH 6.7, aqueous solution of 5 mM ferrozine, and solutions of AG or AS extracts in EtOH (concentrations ranging between 10 μg/mL and 200 μg/mL) were prepared. A total of 4 μL of AG or AS extract solution was added to ammonium iron(II) sulfate hexahydrate in an ammonium acetate buffer (200 μL), incubated at 37 °C for 10 min, and the absorbance (562 nm) was measured. Then, 4 μL of the ferrozine solution was added, incubated again at 37 °C (10 min), and the absorbance at 562 nm was measured. EtOH was used in the control experiments. EDTA was used as a reference iron(II) chelator. The percentage of iron(II) chelation (% Fe(II) chelation) was determined as follows:

Fe(II)_chelating activity_ (%) = [(ABS_CTRL_ − ABS_sample_)/ABS_CTRL_] × 100 (3)

where ABS_CTRL_ and ABS_sample_ are the absorbance (562 nm) of the control and sample, respectively. Dose–response curves allowed the IC_50_ values calculation, using Graph-Pad Prism 9 software (GraphPad Inc., San Diego, CA, USA).

#### 3.4.2. Cell Cultures

The cell lines employed in the experiments were obtained from American Type Culture Collection (ATCC, Manassas, VA, USA). Human mammary epithelial cell line MCF-10A and RAW 264.7 murine macrophages were cultured as previously described [43], at 37 °C, in a humidified atmosphere, 5% of CO_2_.

#### 3.4.3. MTT Assay

Cells were grown in complete medium, then treated and viability was assessed using the 3-(4,5-dimethylthiazol-2-yl)-2,5-diphenyltetrazolium bromide reagent (MTT, Sigma–Aldrich, Milan, Italy), as described in [44]. For each sample, the absorbance, measured at 570 nm, was expressed as a percentage of the control, and IC_50_ values were determined using GraphPad Prism 9 software (GraphPad Inc., San Diego, CA, USA). Mean values and standard deviations (SD) of three independent experiments were reported.

#### 3.4.4. Griess Assay

The Griess assay was adopted for measuring the NO production on murine macrophages RAW 264.7, as reported by [43]. Shortly, cells were plated in 48 multi-wells, treated with the extracts or xanthan gel formulation, at the proper concentrations and time and, then stimulated with the lipopolysaccharide (LPS, Sigma Aldrich, Milan, Italy) at the final concentration of 1 µg/mL. Cell medium was mixed with the Griess reagent (Sigma Aldrich, Milan, Italy) with a 1:1 ratio, left under agitation for 30 min and the absorbance (540 nm) was measured. The NO production (%) was deducted from the absorbance values.

#### 3.4.5. Immunofluorescence Assays

RAW 264.7 cells were grown on glass coverslips in full medium, then treated with DMSO (vehicle, CTRL), LPS (1 µg/mL), or co-treated with LPS and AS extract (AS, 1000 µg/mL) or Indomethacin (Ind, 280 µg/mL, positive control) for 24 h. Next, cells were washed with PBS and fixed using cold methanol (15 min, −20 °C). *i*NOS and NF-kB primary antibodies (Santa Cruz Biotechnology Inc., CA, USA) diluted in BSA 2%, were added to the fixed cells and incubated overnight at 4 °C) as already described [45]. The secondary antibody Alexa Fluor^®^568 conjugate goat-anti-mouse (1:500 dilution, Thermo Fisher Scientific, Waltham, MA, USA) was adopted. A 0.2 µg/mL DAPI solution (Sigma Aldrich, Milan, Italy) was used for nuclei counterstain, incubating for 10 min at room temperature, protected from light. Images were visualized by the means of a Leica DM 6000 fluorescence microscope and Leica Application Suite X (LAS X) software (version 3.7.4, Leica Microsystems, Wetzlar, Germany) was used for image acquisition and processing. ImageJ software (version 1.54r, National Institutes of Health, Bethesda, MD, USA) was employed for fluorescence quantification.

#### 3.4.6. Wound-Healing Assay

MCF-10A cells were plated on 12-well plates, in full medium, until the production of confluent monolayers. Next, the monolayers were scratched with a pipette tip to form a wound and incubated with full medium supplemented with AG or AS extracts or control (DMSO), as previously described [46]. Images at time zero (t 0h) and at two different times, 24 and 48 h (t end), were acquired to record the initial wound area and the wounded monolayer closure due to cell migration, respectively. An inverted microscope equipped with a digital camera (Leica DM6000, Leica Microsystems, Wetzlar, Germany, 5× magnification) was employed for acquiring the images. Cells migration was expressed as a percentage of wound closure, according to the following Equation (4):

Wound closure % = [A(t 0h) − A(t end)]/A(t 0h)(4)

DMSO (vehicle) was used as a control. Images were analyzed using LAS X software and the wound areas measured by ImageJ. Each experiment was performed in triplicate (representative images are shown).

### 3.5. Xanthan Gum-Based Emulgels

#### 3.5.1. Materials and Preparation of Xanthan Gum-Based Emulgels

Emulgels (50 g) structured with xanthan gum (Farmalabor Srl, Canosa di Puglia, Italy) were prepared using a rotor–stator system (UT T50, IKA-Werke, Staufen, Germany), while maintaining a constant composition of 10% *w*/*w* oil phase and 90% *w*/*w* aqueous phase. All formulations are reported in Table 1. The oil phase was prepared by dissolving 0.5% *w*/*w* soy lecithin (based on the weight of oil) (Ivovital, Aachen, Germany), used as the emulsifier, in sunflower oil (La Collina d’Oro, Valdidentro, Italy) at 70 °C using a heated magnetic stirrer. This lecithin concentration was selected based on a previous study in which this value was identified as the critical micelle concentration (CMC) [47]. The aqueous phases, with different concentrations of xanthan gum (XG), were homogenized for 1 min at 2000 rpm and subsequently for another 1 min at 4000 rpm. The oil phase was then added, and mixing was continued for an additional 2 min at 4000 rpm.

#### 3.5.2. Preparation of Emulgels Containing Aloe Extract

The emulgels exhibiting characteristics most similar to the reference formulations were selected among those investigated, and aloe gel (AG) and skin (AS) extracts were incorporated into these systems. The extract was dispersed in the aqueous phase prior to the addition of the gelling agent (xanthan gum) using an Ultra-Turrax homogenizer for 2 min at 2000 rpm. Subsequently, the appropriately weighed gelling agent was added, and the material homogenized as described in the previous section.

The concentration of extract incorporated into the emulgel was selected taking into account the dilution required for subsequent cell-based assays. Indeed, a concentration of 10 mg/mL was chosen for both extracts. This value also represents the highest extract content that could be incorporated; in fact, at a higher concentration, the emulgel becomes heterogenous and granular, loosing stability. The emulgel samples loaded with aloe extracts are reported in Table 1.

#### 3.5.3. Rheological Characterization

All samples were stored for 24 h at 4 °C before carrying out the characterization. The rheological characterization of benchmark samples and emulgels was performed with Small Amplitude Oscillations (SAOS) in the linear viscoelastic region (LVR) and Flow Curves using a controlled stress rheometer (Haake MARS III, Thermo Fisher Scientific, Waltham, MA, USA) equipped with parallel plates geometry (diameter 50 mm, gap 1.2 ± 0.2 mm). Frequency sweep tests within LVR (previously determined with stress sweep tests conducted between 0.01 and 100 Pa) were performed between 0.1 and 10 Hz at 25 °C. The results are shown in terms of mean and standard deviation. A waiting time of 200 s was applied for all samples, as previously established by time sweep tests performed within the linear viscoelastic region.

Flow curves at 25 °C were carried by varying the shear rate from 0.01 s^−1^ to 100 s^−1^, and a power-law model, Equation (5), was adopted to describe the flow behavior:

(5) $$\eta =k\cdot {\left(\dot{\gamma }\right)}^{n-1}$$

where *k* (Pa s) is the consistency index and *n* is the flow index.

### 3.6. Statistical Analysis

Statistical analysis of variance (one-way ANOVA) followed by Tukey’s multiple-range test were performed for all the biological tests using Prism GraphPad version 9.0 software, establishing the significance at *p* < 0.05 (*), *p* < 0.01 (**), *p* < 0.001 (***), and *p* < 0.0001 (****). Student’s *t*-test was applied to evaluate the statistical significance of a comparison between two independent samples (AG vs. AS) in Table 3. Values are expressed as the mean ± standard deviation (SD) of three different experiments carried out in triplicate. One-way analysis of variance (ANOVA) was used to compare the rheological parameters. Differences were considered significant at *p* < 0.05. Data fitting and the ANOVA test were performed using Origin Pro Software (Version 2022, OriginLab Corporation, Northampton, MA, USA).

## 4. Conclusions

In conclusion, a sustainable strategy was successfully established to valorize *Av* epidermal skin, an agro-industrial byproduct discarded during conventional gel extraction, into an innovative formulation for the potential GERD management. The phytochemical profiling revealed that the AS extract exhibited a higher concentration of bioactive compounds, including anthraquinones, aloeresin D, and 3-O-p-coumaroylquinic acid, compared to the inner parenchyma gel. This trend was further corroborated by its higher total phenolic (TPC) and flavonoid content (TFC). This chemical characterization translated into enhanced in vitro biological activities. While both extracts demonstrated good cell-migration capabilities in the scratch assays, the AS extract exhibited, additionally, potent antioxidant capacity across DPPH^•^, ABTS^•+^, and galvinoxyl radical scavenging assays, alongside a strong iron-chelating activity. In addition, in LPS-stimulated RAW 264.7 macrophages, the AS extract produced a dose-dependent reduction in NO production without inducing cytotoxic effects. Moreover, immunofluorescence assays demonstrated that the AS extract exerts its anti-inflammatory action by suppressing the LPS-induced nuclear translocation of NF-κB, with a consequent downregulation of *i*NOS expression. Starting from these biological findings, a stable emulgel system with a xanthan gum matrix was developed, in which *Av* extracts were encapsulated. Rheological characterization demonstrated that the mechanical profile of the extracts-loaded systems matched the behaviors of two established commercial benchmarks, Gaviscon^®^ and GerdOff^®^, used as references. More importantly, cell-based assays validated that the encapsulation process did not alter the extract’s biological potential, as the XG0.4_10AS formulation retained its dose-dependent anti-inflammatory efficacy. In summary, the novelty of this work lies in developing a formulation based on the byproduct skin extract, which delivers good anti-inflammatory properties while maintaining the rheological performance required for mucosal formulations, matching the existing market benchmarks. Further studies will be conducted to support the future development of this AS-loaded emulgel, translating these findings into disease-specific models, ensuring a safe and stable alternative for potential GERD management.

## Figures and Tables

**Figure 1:**
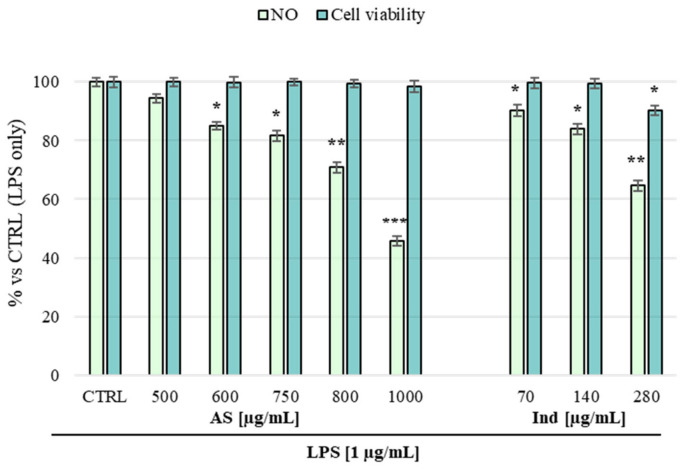
NO production (% vs. CTRL) measured in vitro on murine macrophages RAW 264.7 (light green columns). LPS (1 µg/mL) was used as NO production inducer. AS extract and indomethacin (Ind) were employed at the indicated concentrations. Cell viability (MTT assay, dark green columns) was expressed as % of cell viability with respect to CTRL (LPS only treatment). Columns ± SD are reported (*n* = 3). Treated vs. CTRL, * *p* < 0.05, ** *p* < 0.01, *** *p* < 0.001, or not significant where not indicated.

**Figure 2:**
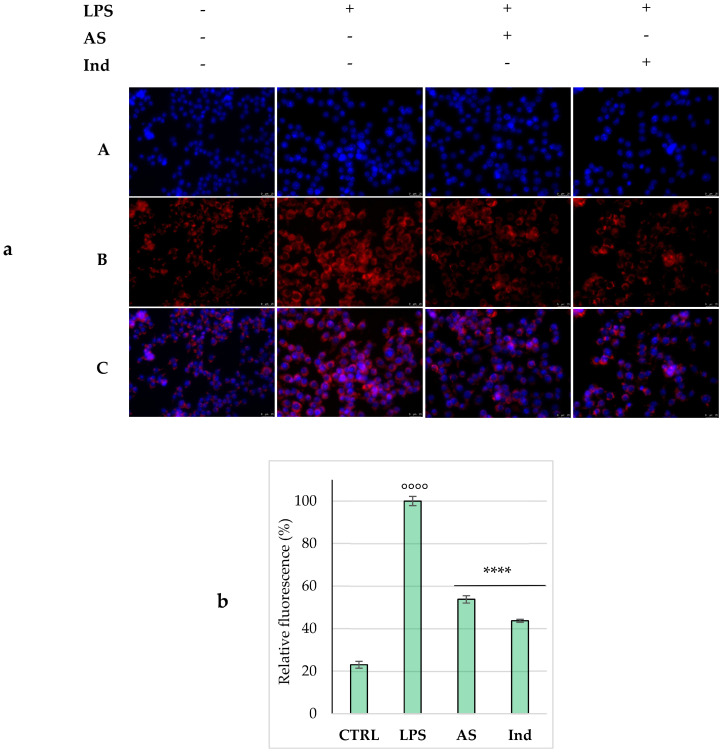
Effect of AS extract on *i*NOS expression in RAW 264.7 macrophages. (**a**) Representative immunofluorescence images of RAW 264.7 cells treated for 24 h with DMSO (vehicle, CTRL), LPS (1 µg/mL), or co-treated with LPS and AS extract (AS, 1000 µg/mL) or Indomethacin (Ind, 280 µg/mL, positive control). Panels (**A**), cell nuclei stained with DAPI (λ_ex/em_ = 350/460 nm); Panels (**B**), *i*NOS protein expression (λ_ex/em_ = 644/665 nm); Panels (**C**), overlay. (**b**) Quantification of relative *i*NOS fluorescence intensity (%), normalized to the LPS-treated group. Data are expressed as mean ± SD. °°°° *p* < 0.0001 LPS vs. CTRL; **** *p* < 0.0001 AS and Ind vs. LPS.

**Figure 3:**
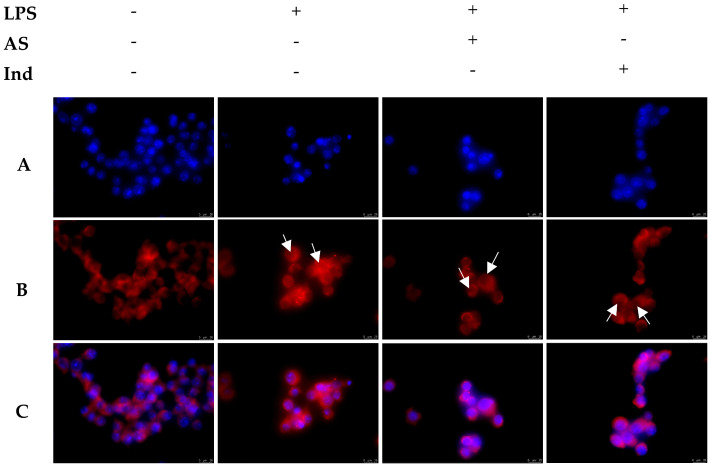
Effect of AS extract on NF-κB nuclear translocation in RAW 264.7 macrophages. Representative immunofluorescence images of RAW 264.7 cells treated for 24 h with DMSO (vehicle, CTRL), LPS (1 µg/mL), or co-treated with LPS and AS extract (AS, 1000 µg/mL) or Indomethacin (Ind, 280 µg/mL, positive control). Panels A, cell nuclei stained with DAPI (λ_ex/em_ = 350/460 nm); Panels B, intracellular localization of NF-κB (λ_ex/em_ = 644/665 nm), with white arrows indicating NF-κB nuclear translocation or not; Panels C, overlay.

**Figure 4:**
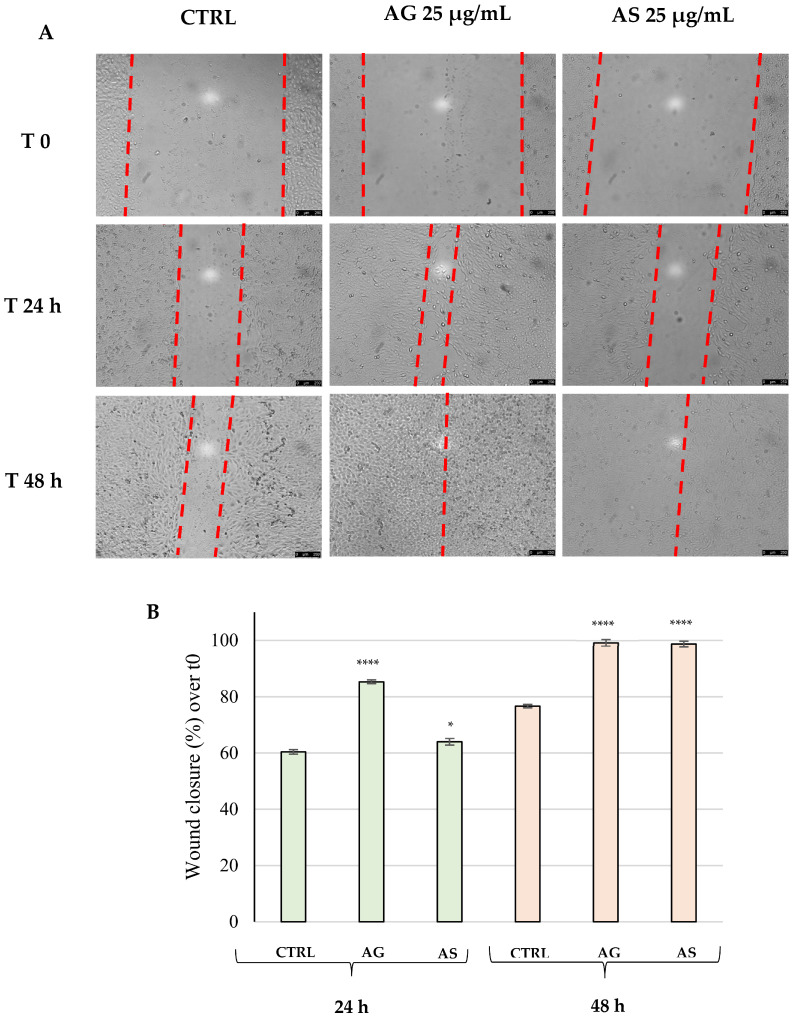
(**A**) Wound-healing assay conducted on MCF-10A cells treated with AG or AS at 25 µg/mL or vehicle (CTRL). Wound closure was assessed after 24 and 48 h of treatment by means of an inverted microscope, at 5× magnification. Dotted red lines highlight the areas lacking cells. (**B**) Wound-closure percentage, at the indicated endpoints, was calculated using ImageJ. * *p* < 0.05 and **** *p* < 0.0001, AG and AS *versus* CTRL.

**Figure 5:**
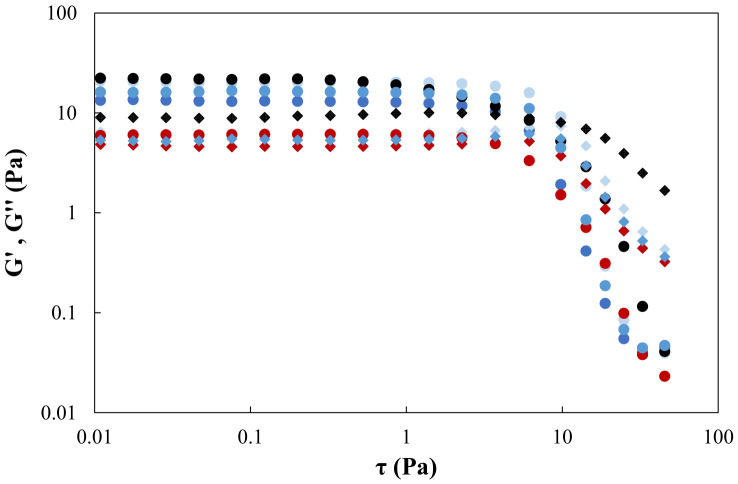
Stress sweep test of reference samples (Gaviscon^®^, black symbol and GerdOff^®^, red symbol) and xanthan gum gel XG0.4 (dark blue symbol), XG0.45 (blue symbol), XG0.5 (light blue symbol). Storage modulus (G′, circles) and loss modulus (G″, diamonds).

**Figure 6:**
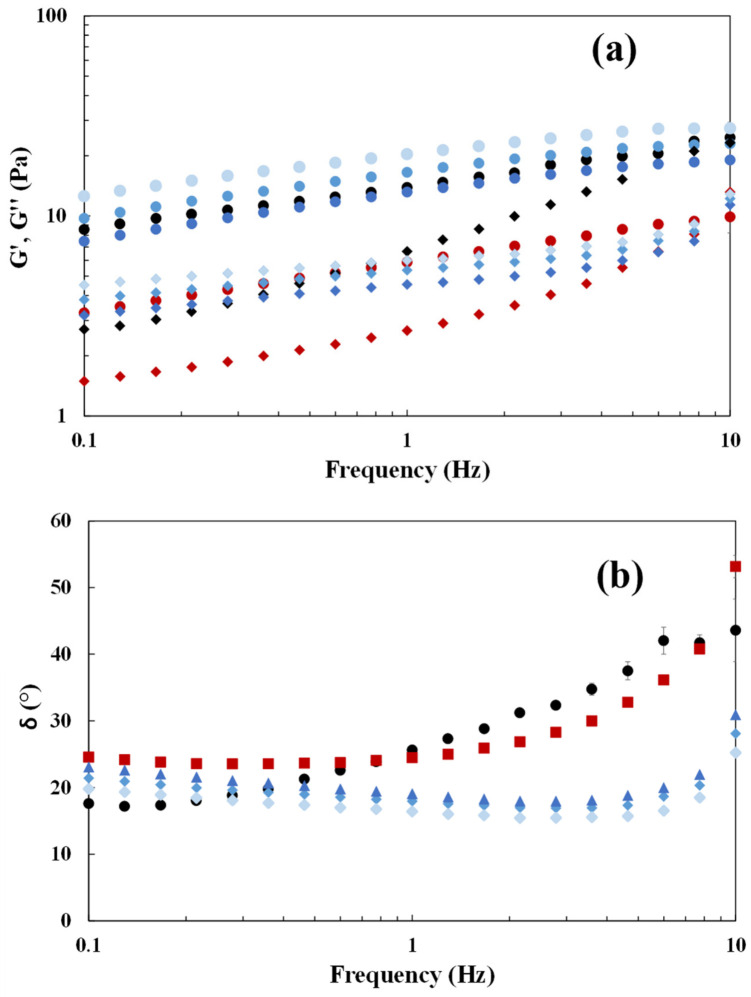
Frequency sweep test of reference samples (Gaviscon^®^, black symbol and GerdOff^®^, red symbol) and xanthan gum gel XG0.4 (dark blue symbol), XG0.45 (blue symbol), XG0.5 (light blue symbol). (**a**) Storage modulus ($G^{\prime }$, circles) and loss modulus ($G^{\prime \prime }$, diamonds), (**b**) phase angle (δ).

**Figure 7:**
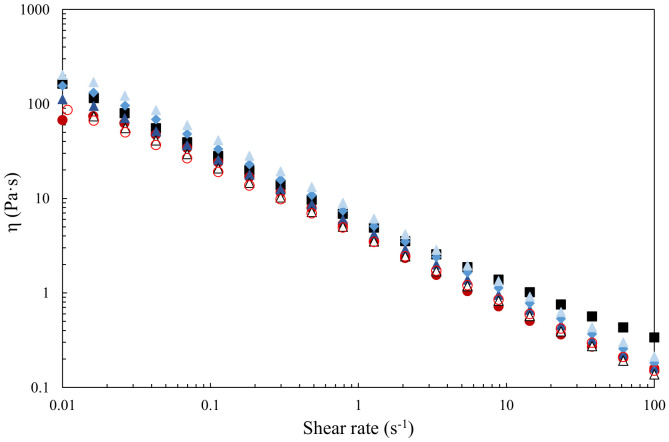
Viscosity curve of reference sample and xanthan gum gel. Gaviscon^®^ full black squares, GerdOff^®^ full red circles, XG0.4 full dark blue triangles; XG0.45 full blue diamonds; XG0.5 full light blue triangles; XG0.4_10AG empty black triangles; XG0.4_10AS empty red circles.

**Figure 8:**
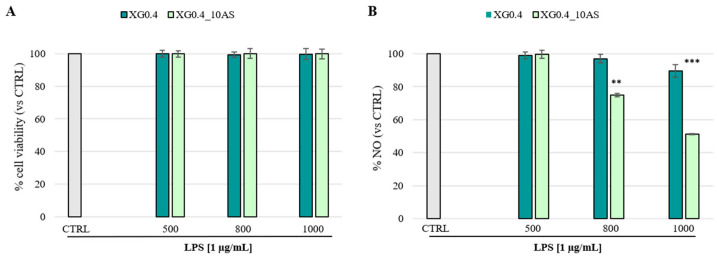
(**A**) Cell viability and (**B**) NO production (%) measured in vitro on RAW 264.7 macrophages. Cells were treated with XG0.4 (light green columns) or XG0.4_10AS (dark green columns), at the indicated concentrations for 24 h, and LPS (1 µg/mL) was used to stimulate NO production. Cell viability was expressed as % with respect to LPS-only treatment (CTRL). Columns ± SD are reported (*n* = 3). Treated vs. CTRL, ** *p* < 0.01, *** *p* < 0.001, or not significant where not indicated.

**Table 1:** Phenolic compound concentrations in *Av* extracts (mg g^−1^ extract) ^ψ^.

Compound	AS	AG	Sign.
3-O-*p*-Coumaroylquinic acid	0.850 ± 0.153	0.198 ± 0.034	**
Aloin B	2.992 ± 0.359	0.023 ± 0.003	**
Aloin A	4.100 ± 0.861	0.042 ± 0.010	**
Aloeresin D	1.906 ± 0.286	0.199 ± 0.028	**
Aloe emodin	tr	0.120 ± 0.024	**

^ψ^ Data are reported as mean ± standard deviation (SD) of three experiments (*n* = 3). tr = trace amount below the limit of quantification. ** *p* < 0.01.

**Table 2:** Total phenolic and flavonoid content.

Sample	TPC ^1^	TFC ^2^
AS	60.75 ± 5.65	3.82 ± 0.07
AG	18.33 ± 1.15	0.48 ± 0.05

^1^ Total phenolic content. ^2^ Total flavonoid content. Data are expressed mean ± SD (*n* = 3). The results are expressed as mg equivalents of chlorogenic acid (TPC) or quercetin (TFC) per g of dry extract.

**Table 3:** In vitro antioxidant activity of *A. vera* leaf extracts ^ψ^.

	IC_50_ (µg/mL)			
	DPPH^•^	ABTS^•+^	GO Assay^•^	Fe (II) Chelating Activity
AG	>1000 ^b^	756.83 ± 73.47 ^b^	>1000 ^b^	>1000 ^b^
AS	619.67 ± 70.59 ^a^	156.90 ± 13.05 ^a^	530.00 ± 16.52 ^a^	66.89 ± 11.32 ^a^
	****	***	***	*
Trolox	6.46 ± 0.11	4.97 ± 0.04	1.26 ± 0.26	-
EDTA	-	-	-	0.77 ± 0.02

^ψ^ Data are expressed as mean ± SD (*n* = 3). Different letters (a or b), for the same parameter, refer to significantly different values. * *p* < 0.05, *** *p* < 0.001, **** *p* < 0.0001.

**Table 4:** Results of rheological characterization: Complex modulus $G^{*}$ (Pa) and phase angle δ (°) at 1 Hz; flow curve fitting: consistency index $k(Pa\cdot s^{n})$ and flow index *n* (-). Different letters, for the same parameter, refer to significantly different values.

ID	Xanthan Gum Fraction $x_{g}$ (*w*/*w*)	Extract Concentration (mg/mL)	Source of the Extract	$G^{*}$ $\left(Pa\right),1\;Hz$	δ $\left({}^{\circ}\right),1\;Hz$	k $\left(Pa\cdot s^{n}\right)$	n (−)
Gaviscon^®^	-	-	-	15.4 ± 0.2 ^c^	25.6 ± 0.2 ^a^	6.1 ± 0.4 ^c^	0.31 ± 0.04 ^bc^
GerdOff^®^	-	-	-	6.5 ± 0.2 ^g^	24.5 ± 0.3 ^b^	5.2 ± 0.2 ^d^	0.312 ± 0.004 ^bc^
XG0.4	0.004	-	-	13.9 ± 0.2 ^d^	19.06 ± 0.03 ^e^	5.8 ± 0.2 ^c^	0.317 ± 0.008 ^abc^
XG0.45	0.0045	-	-	17.4 ± 0.2 ^b^	17.9 ± 0.1 ^f^	7.1 ± 0.2 ^b^	0.286 ± 0.007 ^cd^
XG0.5	0.005	-	-	21.2 ± 0.4 ^a^	16.4 ± 0.1 ^g^	8.4 ± 0.2 ^a^	0.268 ± 0.003 ^d^
XG0.4_10AG	-	10	Gel	7.4 ± 0.1 ^f^	23.82 ± 0.02 ^c^	4.6 ± 0.1 ^e^	0.349 ± 0.007 ^a^
XG0.4_10AS	-	10	Skin	8.7 ± 0.1 ^e^	22.18 ± 0.01 ^d^	4.9 ± 0.2 ^de^	0.336 ± 0.009 ^ab^

## Data Availability

The original contributions presented in this study are included in the article.

## Abbreviations

AG	inner parenchyma
AS	epidermal tissue
GERD	Gastro-Esophageal Reflux Disease
*Av*	*A. vera*
COX-2	cyclooxygenase-2
*i*NOS	inducible nitric oxide synthase
PPARγ	peroxisome proliferator-activated receptor-gamma
11β-HSD1	11β-hydroxysteroid dehydrogenase
VEGF	Vascular Endothelial Growth Factor
ROS	Reactive Oxygen species
DPPH	2,2-diphenyl-1-picrylhydrazyl
ABTS	2,2′-azino-bis(3-ethylbenzothiazoline-6-sulfonic acid)
GO	galvinoxyl
EDTA	ethylenediaminetetraacetic acid
LPS	lipopolysaccharide
NOS	nitric oxide synthase
NSAID	nonsteroidal anti-inflammatory drug
MTT	3-(4,5-dimethylthiazol- 2-yl)-2,5-diphenyltetrazolium bromide reagent
SD	standard deviations
SAOS	Small Amplitude Oscillations
LVR	linear viscoelastic region
